# Invasive carcinoma segmentation in whole slide images using MS-ResMTUNet

**DOI:** 10.1016/j.heliyon.2024.e26413

**Published:** 2024-02-19

**Authors:** Yiqing Liu, Huijuan Shi, Qiming He, Yuqiu Fu, Yizhi Wang, Yonghong He, Anjia Han, Tian Guan

**Affiliations:** a Institute of Biopharmaceutical and Health Engineering, Tsinghua Shenzhen International Graduate School, Shenzhen, Guangdong, China; b Department of Pathology, the First Affiliated Hospital of Sun Yat-sen University, Guangzhou, Guangdong, China

**Keywords:** Invasive carcinoma segmentation, Whole slide image, Transformer, Conditional random field, Multi-task learning

## Abstract

Identifying the invasive cancer area is a crucial step in the automated diagnosis of digital pathology slices of the breast. When examining the pathological sections of patients with invasive ductal carcinoma, several evaluations are required specifically for the invasive cancer area. However, currently there is little work that can effectively distinguish the invasive cancer area from the ductal carcinoma in situ in whole slide images. To address this issue, we propose a novel architecture named ResMTUnet that combines the strengths of vision transformer and CNN, and uses multi-task learning to achieve accurate invasive carcinoma recognition and segmentation in breast cancer. Furthermore, we introduce a multi-scale input model based on ResMTUnet with conditional random field, named MS-ResMTUNet, to perform segmentation on WSIs. Our systematic experimentation has shown that the proposed network outperforms other competitive methods and effectively segments invasive carcinoma regions in WSIs. This lays a solid foundation for subsequent analysis of breast pathological slides in the future. The code is available at: https://github.com/liuyiqing2018/MS-ResMTUNet

## Highlights

• We introduce Transformer architecture in IC segmentation, combining it with CNN for a dual-branch encoder.
• We consider other categories prone to confusion with IC, using a classification branch for effective differentiation.
• We incorporate multi-scale and CRF techniques to enhance WSI inference in our model.

## 1 Introduction

Breast cancer (BC) is the most frequently diagnosed malignancy and the leading cause of cancer-related deaths among women [1]. The incidence and mortality of BC is on the rise worldwide, particularly in low-resource countries. The gold standard for the diagnosis of BC is histopathological examination, which involves a pathologist visually evaluating biopsy material on microscopic slides to determine the appropriate treatment for the patient [2]. However, the histopathological assessment of morphological features is subjective and the high workloads of pathologists may increase the risk of misdiagnosis. In recent years, deep learning has advanced rapidly and has made significant progress in the field of pathological image analysis [3], [4], [5], [6], [7], [8]. These advancements suggest that computer-aided pathological diagnosis systems have the potential to make a positive clinical impact and will likely be increasingly integrated into the daily work of pathologists in the future.

The most common type of BC is invasive carcinoma (IC), which can spread from the ducts or lobules to the surrounding tissue. When examining the pathological sections of patients with IC, several evaluations are required specifically for the invasive cancer area. Therefore, the accurate identification of invasive carcinoma is crucial in breast pathological diagnosis. For a computer-aided diagnosis system, automatic whole slide image (WSI) analysis can only be successful if the IC area can be automatically detected. Otherwise, the system's utility would be greatly reduced as the pathologist would have to manually exclude non-invasive tumor regions such as DCIS regions when using the system. At present, many deep learning techniques have been developed for quantitative immunohistochemical assessment [9], [10], [11], cancer grading [12], [13], TILs scoring [14], [15], and other pathology evaluations. However, these works have yet to fully automate the processing of WSIs.

IC segmentation is a challenging task that involves two main challenges. Firstly, relying on a single feature can lead to misdiagnosis, necessitating the integration of multiple features for accurate segmentation. For example, the lack of significant morphological differences between IC and DICS complicates their differentiation. To address this issue, the model must be capable of capturing the low-magnification structural features of the IC area as well as the characteristics at the boundaries between the IC area and surrounding tissues. Moreover, the situation is further complicated by the potential for confusion between areas of benign pathology or pre-cancerous lesions and invasive carcinoma. Secondly, the segmentation of IC must take into account large-scale features and contextual interactions. Due to the considerable variability in the size of IC areas, models with a fixed input size can easily result in inconsistent semantic segmentation when processing WSIs. To overcome this, the model needs to process features at various scales and capture the global context of the IC area. This helps the model to better understand the relationship between the IC area and the surrounding tissue, thereby improving the consistency of semantic segmentation.

Despite the current research on tumor region segmentation in breast tissue pathological images, the segmentation of IC remains a challenging and unresolved problem. Ho et al. [16] have segmented various types in breast tissue slides but have not further distinguished the tumor category into DCIS and IC. Ni et al. [17] specifically differentiate between DCIS and IC but do not consider categories such as pathological benign and pre-cancerous lesions, which can be easily confused with IC in practical scenarios. Huang et al. [18] consider normal lobules and benign lesions, but they use a small amount of data, leaving significant room for improvement in performance. Furthermore, the existing research models used for this task are all based on CNN architectures. The performance of Transformer models [19], [20] for IC segmentation has not been investigated. These models have been increasingly used in computer vision tasks in recent years. Thanks to their self-attention module, they excel at capturing contextual information in images, showing tremendous potential for IC segmentation tasks.

To achieve accurate invasive carcinoma region segmentation in breast tissue pathological images, we propose Residual Mix Transformer Unet (ResMTUnet). This model not only has the capability to accurately classify the type of input image (normal, abnormal, or invasive carcinoma), but also effectively segment the corresponding contour. ResMTUnet is based on an encoder-decoder architecture, where it combines the ability to model both global features utilizing vision transformer and local features utilizing CNN. Additionally, it employs multi-task learning to enhance the performance of both segmentation and classification tasks. Furthermore, in order to accomplish invasive carcinoma automatic recognition and segmentation in a WSI, we design a novel transformation method that can convert the trained ResMTUnet into a multi-scale input model, and adopt conditional random field to fuse multiple scale outputs.

In general, our contributions are as follows:

• We emphasize the importance of Transformer in the IC segmentation task and introduce this architecture for the first time in IC segmentation. By combining it with CNN in a dual-branch encoder, we aim to better capture the features of IC from multiple dimensions and improve the accuracy of segmentation.
• In addition to DCIS, we also consider other categories that are prone to confusion with IC, enhancing the model's usability in practical scenarios. To better differentiate these categories, we introduce a classification branch, which provides information enhancement through multi-task learning strategies.
• To improve the model's inference on WSIs, we incorporate it with multi-scale strategy and conditional random field (CRF). The multi-scale and CRF components are decoupled from the ResMTUnet, allowing the model to adapt to both single-scale and multi-scale inference scenarios.

## 2 Related works

### 2.1 Histopathological image classification

Many digital pathology techniques have been proposed for histopathological image classification:

**Traditional machine learning method.** These methods are inspired by the diagnostic process of pathologists, which typically involves hand-crafted features associated with the lesion. These features are integrated with machine learning models, such as SVM, decision trees, and random forests to classify pathological images. Different types of hand-crafted features are often combined to improve the model performance. For example, Doyle et al. used an SVM classifier to differentiate cancerous and non-cancerous images by inputting texture and nuclear structure features [21]; Yamamoto et al. classified histological types of intraductal proliferative lesions based on subtle morphological differences of microenvironmental myoepithelial cell nuclei [22]; Aswathy et al. proposed an integrated feature extraction module for extracting and merging three groups of biologically important and clinically valid features (texture, geometric and color features) from breast cancer histopathology images [23].

**CNN-based method.** In recent years, Convolutional Neural Network (CNN) has become the most widely-used deep learning method for pathological image analysis tasks. CNNs possess a significant advantage in image processing and classification due to their ability to automatically extract and learn meaningful features from images. This makes them particularly effective in handling high-dimensional and noisy data. For instance, Roy et al. proposed a patch-based CNN classifier for the automatic classification of histopathological breast images [24]. Jiang et al. introduced a small SE-ResNet module to the CNN for breast cancer histopathological image classification [25]. Vuong et al. proposed a multi-scale method, which can recognize and utilize patterns at multiple scales in deep neural networks, and therefore used as a basis to classify pathological images [26]. Hameed et al. employed the multi-level features of deep CNNs to conduct multi-class classification of breast cancer histopathological images [27].

**Transformer-based method.** Recent studies [19], [20] have extended the transformer model [28] to visual tasks. These methods have been shown to be effective in learning global representations, and the Vision Transformer (ViT) architecture in particular has been gaining popularity in the field of pathological image analysis due to its use of self-attention module to model context relationships within images. For example, Mehta et al. introduced HATNet, a transformer-based method that uses self-attention to hierarchically aggregate information at different levels of the model, allowing for the learning of spatial relationships between words and bags [29]. Chen et al. proposed the Hierarchical Image Pyramid Transformer (HIPT) architecture, which utilizes the natural hierarchical structure of WSIs and employs two levels of self-supervised learning to learn high-resolution image representations [30]. Additionally, Stegmüller et al. proposed ScoreNet, an efficient transformer-based architecture for organ pathology image classification that combines fine-grained local attention mechanism and coarse-grained global attention module to extract features at both the cellular and tissue level [31].

**Graph-based method.** Although CNNs have proven successful in image classification and feature representation, graph-based organizational pathology analysis is a promising alternative. Graph representations of pathological images can effectively describe the composition of tissue by incorporating morphological, topological, and biologically meaningful interactions between entities. This allows for the incorporation of pathological and task-specific prior knowledge when constructing “meaningful” tissue representations. In recent research, Anand et al. [32] have utilized graph convolutional networks (GCNs) to classify cancer by modeling images from tissue sections as multi-attribute graphs of their component cells. Zhou et al. [33] proposed a new cell graph convolutional neural network (CGC-Net) to convert large histological images into graphs for colon cancer grading tasks. Wang et al. [34] introduced a weakly supervised method using GCN to grade tissue microarrays (TMA) by modeling the spatial organization of cells as graphs to better capture the proliferation and community structure of tumor cells. Pati et al. [35] proposed a new multi-level entity graph representation of tissue samples to model the hierarchical structure of encoded tissue entities and their interactions.

### 2.2 Histopathological image segmentation

The goal of semantic segmentation is to divide a given image into several visually meaningful or semantically relevant regions for subsequent image analysis and visual understanding. This task plays an important role in medical image analysis, enabling precise and accurate identification of structures and regions of interest within images. In recent years, deep learning methods have produced a new generation of segmentation models with significant performance improvements, becoming the mainstream solution for semantic segmentation. For example, Long et al. [36] proposed the Fully Convolutional Network (FCN), which can perform end-to-end training, while Ronneberger et al. [37] proposed the U-Net for biomedical image segmentation, which has become a classic framework for medical image segmentation. Additionally, Chen et al. proposed the DeepLabV3+ segmentation model [38], which incorporates atrous convolution and ASPP modules to increase the receptive field and better capture context information within images.

With the introduction of Transformer models in the field of computer vision, many works have combined the localization capabilities of CNNs with the global awareness of Transformers to produce hybrid models. For example, TransUnet proposed by Chen et al. [39] enhances the locality of convolutional operations by using a transformer encoder that operates on feature maps created by the encoding convolutional network. This hybrid CNN-Transformer architecture preserves the advantages of both transformer and CNN models, utilizing CNNs' rich feature maps and the global context encoded by Transformers to achieve better performance in medical image segmentation.

Several attempts have been made to segment breast cancer regions in recent years [40], [41], [42], [43]. These studies mainly rely on CNNs, however, they fail to distinguish between DCIS and IC. In particular, Cruz-Roa et al. [43] proposed a classification method using ConvNet to detect the presence and degree of invasive breast cancer in whole-slide digital pathology images. The work also mentions the issue of in situ cancer regions possibly being incorrectly classified as invasive cancer. To tackle the challenges of semantic segmentation in breast biopsy, Mehta et al. [44] proposed a new multi-resolution encoder-decoder architecture that enhances the model's ability to capture context information through multi-resolution input. This approach allows for the segmentation of various organ structures in tissue pathology images and its multi-resolution input structure provides a promising solution for distinguishing in situ carcinoma and invasive carcinoma.

## 3 Method

### 3.1 Model architecture

**Figure 1:**
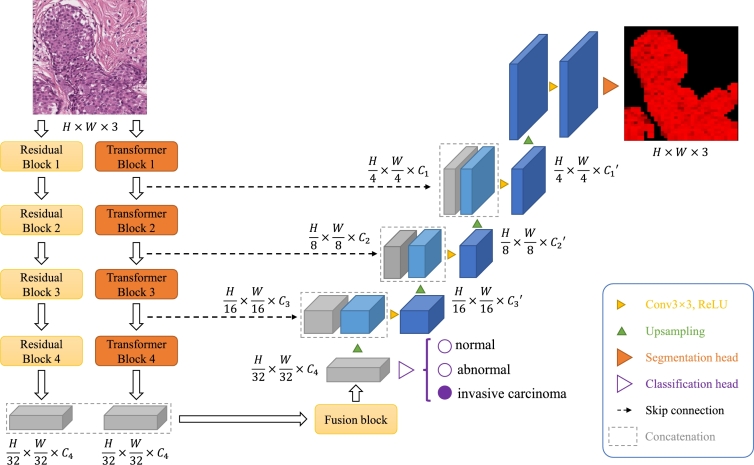
Model architecture of ResMTUNet.

In this part, we introduce our proposed architecture for invasive carcinoma discrimination and segmentation, ResMTUNet. As shown in Fig. 1, ResMTUNet comprises of two key components: (1) a CNN-Transformer encoder, designed to extract multi-level features from the input image; (2) a U-Net structured decoder that fuse these multi-level features to generate aggregate feature maps for semantic segmentation; (3) a classification head and a segmentation head, used to obtain the classification and segmentation results of the image respectively.

Given an image of size H×W×3, we first input it into a CNN-Transformer hybrid encoder to obtain multi-level features at resolutions of {1/4,1/8,1/16,1/32} of the original image resolution. After obtaining these features, we then input the last layer's features of the encoder into a classification head to obtain the category prediction result for the image. Simultaneously, we pass these multi-level features to a U-Net [37] decoder, which is a type of encoder-decoder architecture that is commonly used in image segmentation tasks. The U-Net decoder then aggregates these features into one and sends it to a segmentation head to generate a segmentation mask at a resolution of $H\times W\times N_{class}$, where $N_{class}=4$, representing the number of classes in the segmentation task. Next, we will introduce the design of the encoder and decoder in more detail.

#### 3.1.1 Encoder

The Encoder module extracts features from the input image and generates a series of representations of the image at different scales. The module is composed of both a transformer branch and a residual branch. The output image first passes through the Transformer branch, where a hierarchical feature representation is obtained. The residual feature is then fused with the feature obtained from the last block of the transformer branch through a convolutional fusion module to obtain an enhanced feature. This enhanced feature is subsequently used as input for the classification head to predict the class of the image. Additionally, this feature is also inputted into the decoder, along with features obtained from other blocks of the transformer branch.

The transformer branch consists of four blocks. Each block includes a patch embedding module and a sequence of submodules. The patch embedding module encodes the input image using sliding convolution. For transformer block i∈[1,4], the output of the patch embedding module within the block is $\frac{H}{2^{i+1}}\times \frac{W}{2^{i+1}}\times C_{i}$, where $C_{i}$ represents the number of channels. We also define K,S, and *P*, where *K* is the patch size, *S* is the stride of the convolution, and *P* is the padding size. For i=1, we set K=7,S=4,P=3; for i=2,3,4, we set K=3,S=2,P=1. This overlapping encoding method ensures that local continuity is maintained around the patch. The submodule sequence further encodes the patch embeddings, and each encoded embedding contains information from other embeddings. The submodule sequence includes several submodules, each of which contains an Efficient Self-Attention (ESA) layer and a Mix-FFN layer [45]. ESA can be formulated by Eq. (1), as follows:

(1) $$Attention(Q,K,V)=Softmax(\frac{QK^{T}}{\sqrt{d_{head}}})V.$$

This formula is identical to that of the original self-attention. The only difference between the original self-attention and ESA is the method used to calculate K and V. In order to decrease computational expenses, ESA employs a technique known as “sequence reduction.” The calculation of K and V in ESA can be expressed by Eq. (2) and Eq. (3), as follows:

(2) $$K=W_{k}X^{\prime },V=W_{v}X^{\prime },$$

(3) $$X^{\prime }=Linear(Reshape(X)),$$

where *X* is the input of ESA, Reshape(N/R,C⋅R)(⋅) reshapes X(N,C) into a sequence of shape (N/R)×(C⋅R), $Linear(C_{in},C_{out})(\cdot )$ refers to a linear layer that takes in a $C_{out}$-dimensional tensor as input and generates a $C_{out}$-dimensional tensor as output. Therefore, the dimension of $X^{\prime }$ is N/R×C. As a result, the length of *K* and *V* will also be reduced to 1/R of the original. ESA's output feature is then added with positional encoding using Mix-FFN, which can be expressed by Eq. (4), as follows:

(4) $$x_{out}=MLP(GELU(Conv3\times 3(MLP(x_{in}))))+x_{in},$$

where $x_{in}$ is ESA's output feature and $x_{out}$ is that feature added with positional encoding.

Studies have shown that CNNs excel at capturing local information, while transformers are talented at capturing context information [46]. Combining the two can give the model more powerful representation ability. To achieve this, we introduce a residual branch that works in conjunction with the transformer branch to extract features. This residual branch is composed of a series of residual modules. For the *i*-th (i∈[1,4]) residual convolutional module $ResConv_{i}$, its forward propagation process can be represented by the following formula, Eq. (5):

(5) $$x^{\prime }=ResConv_{i}(x),$$

where *x* and $x^{\prime }$ are the input and output of the convolution module, respectively. We fuse the output of the last convolution module of the residual branch with the output of the last module of the transformer branch using a convolution fusion module. The fused feature will be employed in the subsequent classification and segmentation tasks.

#### 3.1.2 Decoder

The decoder takes a series of features obtained from the encoder and integrates them to produce a feature map for subsequent segmentation task. The decoder consists of multiple convolutional modules. For the *i*-th layer (i∈[1,3]) of the decoder, it receives the output from the *i*-th layer of the encoder and the output from the i+1-th layer of the decoder, denoted as $x_{i}$ and $x_{i+1}^{\prime }$, respectively, as input and generates output $x_{i}^{\prime }$. The computation of $x_{i}^{\prime }$ is given by Eq. (6):

(6) $$x_{i}^{\prime }=Conv(Concat(x_{i},Upsample(x_{i+1}^{\prime }))),$$

where Conv represents convolution operation, Concat denotes concatenation operation, and Upsample represents 2× upsampling. The output from the first layer of the decoder (i.e., $x_{1}^{\prime }$) is fed into the segmentation head before 2× upsampling and convolution operation.

#### 3.1.3 Classification and segmentation heads

The classification head takes the output from the last layer of the encoder as input and outputs a prediction of the image's category, while the segmentation head receives the output from the last layer of the decoder and generates pixel-level segmentation results. This approach enables the encoder to capture more general features, leading to improved performance on each task [47].

The classification head of the model is composed of a global average pooling layer and a fully connected layer. The output of this head is then passed through a Softmax activation function, which produces a probability distribution over the $N_{class}$ classes. The segmentation head is composed of two convolutional layers. The first layer transforms the input image into a feature map with dimensions $H\times W\times C_{0}/2$. The second convolution then further processes this feature map, resulting in an output with dimensions $H\times W\times (N_{class}+1)$.

The classification task and the segmentation task use cross-entropy loss and dice loss, respectively. Therefore, the total loss function can be represented as Eq. (7):

(7) $$L_{total}=\lambda L_{class}+L_{seg},$$

where $L_{class}$ represents the loss for classification task, $L_{seg}$ represents the loss for segmentation task, and *λ* is a weight coefficient. We set it to 0.5 in our experiments.

### 3.2 MS-ResMTUNet for segmentation on WSIs

Due to our trained model requiring inputs with the size of 512×512, when applying it to WSI inference, it is necessary to divide a WSI into 512×512 sized patches for processing. This raises the issue of magnification selection. Too high or too low magnification will result in decreased model accuracy, and a single magnification cannot accommodate the wide range of variations in the size of tumor regions. To tackle this problem, we propose an innovative conversion method that transforms the trained model into a multi-scale input model (i.e., MS-ResMTUNet), ensuring more accurate detection and segmentation of IC regions in WSIs.

**Figure 2:**
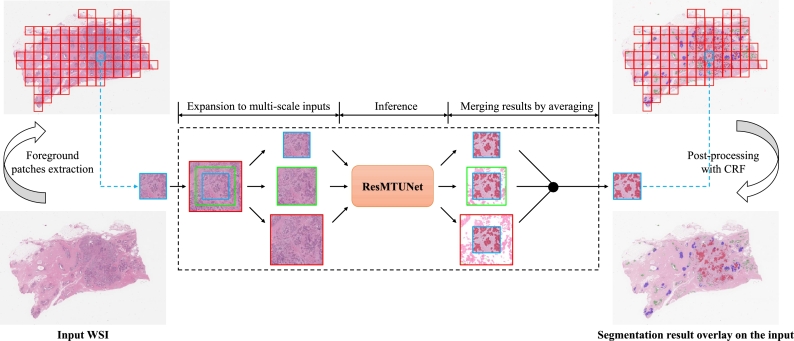
Illustration of MS-ResMTUNet.

The process of segmenting IC regions in a whole WSI using MS-ResMTUNet is shown in Fig. 2. OTSU algorithm is first used to separate the foreground region. Then, the foreground region is divided into several non-overlapping 512×512 sized patches at 20× magnification. For each patch, two center crops are taken based on its position in the WSI, with sizes of 768×768 and 1024×1024, respectively. Then the cropped images are resized to 512×512. These three patches are then fed into the network and three outputs are generated. The last two outputs are scaled back to the original size, cropped to 512×512 pixels in the center, and averaged with the first output to produce a preliminary segmentation result of the patch. After processing all patches, the preliminary segmentation result of the entire WSI is obtained.

To further optimize the segmentation results, we adopt fully connected CRF [48] to post-process the results. This method can capture fine edge details features and effectively utilize long-range dependencies. It uses an energy function, Eq. (8):

(8) $$E(X|I)=\sum_{i}\theta_{i}(x_{i})+\sum_{i,j}\theta_{i,j}(x_{i},x_{j}),$$

where $x_{i}\in X$ represents the label of the *i*-th pixel. The first term on the right is referred to as unary potential. The unary potential is defined as $\theta_{i}(x_{i})=-logP(x_{i})$, where $P(x_{i})$ is the label assignment probability of pixel *i* inferred by MS-ResMTUNet. The second term is referred to as pairwise potential, which has a form that allows efficient inference when using a fully connected graph. Referring to [49], we use Eq. (9):

(9) $$\begin{array}{cc} \theta_{ij}(x_{i},x_{j})=[ & \omega_{1}exp(-\frac{{\left\|p_{i}-p_{j}\right\|}^{2}}{2\sigma_{\alpha }^{2}}-\frac{{\left\|I_{i}-I_{j}\right\|}^{2}}{2\sigma_{\beta }^{2}})\\ + & \omega_{2}exp(-\frac{{\left\|I_{i}-I_{j}\right\|}^{2}}{2\sigma_{\gamma }^{2}})]\mu (x_{i},x_{j}) \end{array},$$

where $\mu (x_{i},x_{j})=1$ if $x_{i}\neq x_{j}$, else zero. This means that only nodes with different labels will be penalized. The pairwise potential consists of two gaussian kernels. The first “bilateral” kernel depends on the pixel position (denoted as *p*) and the input image (denoted as *I*), it forces pixels with similar color and position to have similar labels. The second kernel only depends on the pixel position and penalizes isolated small regions in space, which enforces a more consistent spatial segmentation. The hyperparameters $\sigma_{\alpha }$, $\sigma_{\beta }$, and $\sigma_{\gamma }$ control the scale of the gaussian kernels, which are empirically determined.

### 3.3 Dataset construction

To train the ResMTUNet model, we transformed the BRACS classification dataset [50] into a semantic segmentation dataset by annotating the images with segmentation masks. Given the intricate shape of the tissue contours, we devised a semi-automatic annotation process to reduce the annotation workload. This process consisted of two stages: pre-segmentation and refinement.

**Figure 3:**
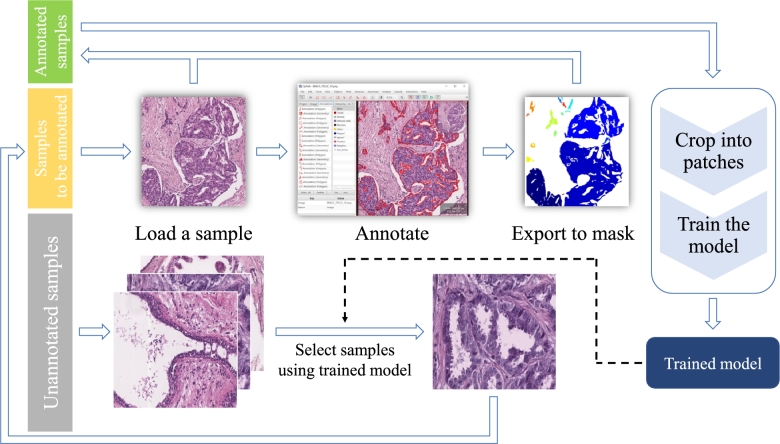
Process of segmentation dataset construction.

The process of pre-segmentation mask generation is shown in Fig. 3. To train the pre-segmentation model, we first extracted a small number of samples from the BRACS training set and annotated the contours of the epithelial tissue using Qupath [51]. The annotations were then exported as masks. The annotated samples and masks were cut into 512×512 size patches, randomly divided into training and validation sets in an 8:2 ratio, and used to train a pre-segmentation model. We choose UNext [52] as our pre-segmentation network due to its lightweight features. The model was then used to perform inference on a portion of the unlabeled samples in a sliding window manner, and samples with poor results were added to the pending annotated samples list for manual annotation. This process was repeated until each unlabeled sample showed good result. Finally, the best performing pre-segmentation model was used to infer all the samples in the BRACS dataset to obtain the final pre-segmentation masks.

In the pre-segmentation model inference stage, 60 samples were manually annotated out of 3657 in the original BRACS training set. To fully utilize the unlabeled data and improve the quality of the mask, the samples and pre-segmentation masks in the training and validation sets were resized to 512×512 and retrained. The best performing model on the validation set was then used to infer all the data and produce the final epithelial segmentation mask. Different class labels were assigned to the segmentation masks of various sample categories to form the final segmentation dataset. In general, the number of samples used for training, validation, and testing were 3657, 312, and 570, respectively.

## 4 Experiments

### 4.1 Experimental setup

#### 4.1.1 Training settings

All networks are trained using the SGD optimizer with a momentum of 0.9 and an initial learning rate of 1e−3. The learning rate is halved every 7 epochs, and the batch size is 16. Each network is trained for 30 epochs, with the backbone of the model initialized with weights pre-trained on the ImageNet dataset. The images in the BRACS dataset are resized to 512×512 for model training and testing, and data augmentation techniques such as horizontal flipping, vertical flipping, Gaussian blurring, and random brightness adjustment are employed during the training process. All networks are implemented on the PyTorch platform and executed on a workstation with 8 A100 GPUs.

#### 4.1.2 Evaluation metrics

In the experiments for the classification task, we utilize 5 evaluation metrics to quantitatively assess the performance of the proposed method and other competing methods: 1) Overall classification accuracy (*Acc*); 2) Arithmetic mean of the per-class F1-scores (*F*1); 3) Arithmetic mean of the precisions for invasive carcinoma samples ($P_{ic}$); 4) Arithmetic mean of the recalls for invasive carcinoma samples ($R_{ic}$); 5) Arithmetic mean of the F1-scores for invasive carcinoma samples ($F1_{ic}$).

In the experiments for the segmentation task, we evaluate the performance using Intersection over Union (IoU) and Dice coefficient as the evaluation indicators. For all samples in the test set, we calculate the average IoU and Dice scores, denoted as *IoU* and Dice, respectively. Additionally, we calculate the average IoU and Dice scores specifically for invasive carcinoma samples in the test set, denoted as $IoU_{ic}$ and $Dice_{ic}$, respectively.

### 4.2 Experiments for classification task

The proposed ResMTUNet is compared with six popular CNNs (ResNet [53], Xception [54], DenseNet [55], EfficientNet [56], SE-ResNet [57] and ConvNeXt [58]), two Transformers (Vision Transformer [19] and Swin Transformer [59]) and two recent approaches (HACT-Net [35], MSBP-Net [26]). As HACT-Net is a type of graph neural network that differs from other competing networks, it adheres to the training configuration in the original paper when conducting classification experiments. Other networks are trained and tested following the same training and testing strategies with the proposed network, except that the input sizes of ConvNeXt and Vision Transformer are set to 224×224.

The quantitative results of breast carcinoma classification are shown in Table 1. The proposed ResMTUNet achieved a score of 90.14% and 89.2% for *Acc* and *F*1, respectively. In distinguishing invasive carcinoma from other subtypes, we obtained a score of 90.11%, 91.11%, and 90.56% for $P_{ic}$, $R_{ic}$, and $F1_{ic}$, respectively. These demonstrate that the proposed network is capable of distinguishing invasive carcinoma from other subtypes. In the comparative experiments, the proposed ResMTUNet was superior to other methods in 5 out of 6 evaluation metrics, as shown in Table 1.

**Table 1:** Evaluation on classification task.

	Acc (%)	F1 (%)	P${}_{ic}$ (%)	R${}_{ic}$ (%)	F1${}_{ic}$ (%)
ResNet34	87.37 ± 0.95	85.77 ± 1.36	88.90 ± 3.84	84.94 ± 4.13	86.75 ± 1.85
ResNet50	87.89 ± 0.12	86.75 ± 0.37	91.70 ± 1.64	84.45 ± 0.68	87.92 ± 1.09
Xception	85.93 ± 1.26	84.23 ± 1.46	90.18 ± 2.92	78.76 ± 1.83	84.07 ± 1.88
DenseNet201	86.95 ± 0.85	85.35 ± 1.49	**93.07 ± 2.00**	77.78 ± 7.36	84.52 ± 4.04
EfficientNet-b4	84.35 ± 0.68	83.57 ± 1.16	89.91 ± 3.90	84.69 ± 3.33	87.17 ± 2.70
EfficientNet-b5	79.09 ± 6.52	75.23 ± 7.90	73.07 ± 14.37	74.81 ± 4.67	73.26 ± 8.98
SE-ResNet101	75.30 ± 0.99	71.05 ± 1.69	90.12 ± 3.46	58.77 ± 6.80	70.86 ± 4.25
SE-ResNet50	86.74 ± 0.88	85.08 ± 1.01	85.42 ± 1.29	86.67 ± 0.55	86.04 ± 0.72
ConvNeXt	87.09 ± 1.27	84.98 ± 2.37	90.01 ± 2.09	78.03 ± 9.26	83.36 ± 5.84
Vision Transformer	86.14 ± 1.52	82.97 ± 2.36	87.08 ± 3.20	71.36 ± 9.93	78.03 ± 5.47
Swin Transformer	87.51 ± 1.11	85.52 ± 1.74	87.54 ± 1.79	81.73 ± 6.62	84.44 ± 4.01
HACT-Net	81.05 ± 1.36	80.29 ± 1.55	84.13 ± 5.65	83.95 ± 4.09	83.88 ± 2.86
MSBP-Net	85.72 ± 0.27	83.49 ± 0.33	88.18 ± 3.32	77.53 ± 4.74	82.38 ± 1.73
ResMTUNet	**90.14 ± 0.90**	**89.20 ± 0.87**	90.11 ± 2.74	**91.11 ± 1.83**	**90.56 ± 0.71**

### 4.3 Experiments for segmentation task

We then compared ResMTUNet to a variety of segmentation models on the BRACS dataset with segmentation annotations. We chose three architectures of segmentation models, U-net, MA-net [60], and DeepLabv3+ [38] with CNNs tested in classification experiments as backbones. The segmentation models and their corresponding training and testing procedures were implemented using SegmentationModels. Since DenseNet encoder is not compatible with DeeplabV3+ architecture and Xception encoder is not compatible with MA-net and DeeplabV3+ architectures, these three combinations were not included in the experiments.

The quantitative results of breast carcinoma segmentation are shown in Table 2. Trained on the segmentation dataset transformed from BRACS, the proposed ResMTUNet achieved a score of 83.49%, 76.20%, 91.04%, and 86.64% for *IoU*, $IoU_{ic}$, Dice, and $Dice_{ic}$, respectively. In the comparative experiments, the proposed ResMTUNet outperformed other segmentation models by >2.24% in *IoU*, >5.69% in $IoU_{ic}$, >0.91% in Dice, and >3.99% in $Dice_{ic}$, showing its strong ability to segment breast carcinoma.

**Table 2:** Evaluation on segmentation task.

Method		IoU (%)	IoU${}_{ic}$ (%)	Dice (%)	Dice${}_{ic}$ (%)	GFLOPs	Params.
U-net	densenet201	81.04 ± 1.04	67.54 ± 2.24	90.13 ± 0.48	80.36 ± 1.99	45.79	28.59M
	efficientnet-b4	62.40 ± 2.66	54.13 ± 1.53	77.41 ± 1.79	70.14 ± 1.27	11.33	2.80M
	efficientnet-b5	65.11 ± 3.12	52.74 ± 1.51	78.78 ± 3.02	68.38 ± 0.60	11.76	3.05M
	resnet34	77.30 ± 3.55	65.84 ± 2.86	86.61 ± 2.08	79.81 ± 2.17	31.51	24.44M
	resnet50	80.38 ± 1.22	67.20 ± 1.52	88.75 ± 0.56	81.39 ± 0.91	42.94	32.53M
	se_resnet101	80.77 ± 2.12	67.36 ± 3.57	89.22 ± 2.05	80.43 ± 2.30	61.28	56.30M
	se_resnet50	78.10 ± 3.27	65.38 ± 3.49	87.05 ± 2.60	79.43 ± 3.02	41.75	35.06M
	xception	79.54 ± 1.11	66.38 ± 1.92	87.71 ± 0.94	79.44 ± 1.88	42.49	28.78M

MA-net	densenet201	79.91 ± 2.40	67.70 ± 1.64	88.82 ± 1.49	80.73 ± 1.17	76.53	133.73M
	efficientnet-b5	61.42 ± 5.44	52.54 ± 3.02	75.99 ± 4.16	68.84 ± 2.62	12.70	10.04M
	resnet50	80.76 ± 1.08	70.51 ± 4.06	89.36 ± 0.66	82.65 ± 2.88	74.73	147.45M
	se_resnet101	81.25 ± 1.66	67.72 ± 3.21	89.65 ± 1.01	80.72 ± 2.27	93.06	171.22M

DeeplabV3+	efficientnet-b5	67.12 ± 0.70	56.17 ± 1.13	80.32 ± 0.50	71.93 ± 0.93	3.05	1.32M
	resnet50	68.97 ± 2.44	55.89 ± 3.21	81.62 ± 1.71	71.66 ± 2.63	36.93	26.68M
	se_resnet101	71.67 ± 1.71	59.45 ± 2.54	83.49 ± 1.16	74.55 ± 2.00	55.67	50.45M

ResMTUNet		**83.49 ± 1.22**	**76.20 ± 1.79**	**91.04 ± 0.92**	**86.64 ± 1.51**	91.13	110.73M

**Figure 4:**
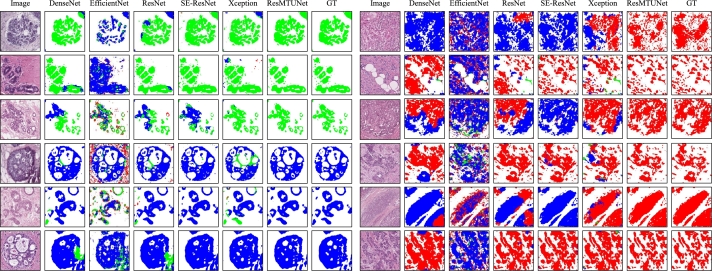
Qualitative comparison results between ResMTUNet and other segmentation models. All segmentation models used for comparison were based on the U-Net architecture, with only their backbone shown in the figure. Red, blue, and green represent three different categories: invasive carcinoma, carcinoma in situ, and normal.

In addition, we show several qualitative comparison results in Fig. 4. As a result, our proposed segmentation method provides the best segmentation results visually, exhibiting high consistency with the ground truth annotations. Other methods, however, can result in semantic discontinuity between adjacent regions with the same type of tissue, and even faulty predictions about the type of tissue in the entire region.

### 4.4 Ablation study

We conducted a comprehensive set of experiments to demonstrate the value of the dual-branch and dual-branch design in ResMTUNet. Table 3 presents the performance metrics of ResMTUNet and two ablation variants on the segmentation and classification tasks in the test set. The ablation variant with the CNN branch removed outperformed the ablation variant with the Transformer branch removed on the metrics for the segmentation task, while the latter outperformed the former for the classification task. ResMTUNet achieved state-of-the-art or near-optimal performance on both tasks simultaneously, demonstrating that ResMTUNet can effectively combine the respective advantages of CNN and Transformer to form a complex with better performance.

**Table 3:** Benefits of the CNN-Transformer encoder and the double-headed structure.

	Acc (%)	F1 (%)	F1${}_{ic}$ (%)	IoU (%)	IoU${}_{ic}$ (%)	Dice (%)	Dice${}_{ic}$ (%)
ResMTUNet	**90.14 ± 0.90**	**89.20 ± 0.87**	**90.56 ± 0.71**	83.49 ± 1.22	**76.20 ± 1.79**	91.04 ± 0.92	**86.64 ± 1.51**
w/o CNN branch	89.68 ± 0.91	88.44 ± 1.20	88.97 ± 2.03	**83.67 ± 1.89**	73.76 ± 2.29	**91.61 ± 0.70**	84.96 ± 1.62
w/o Transformer branch	89.33 ± 0.70	88.67 ± 0.79	90.44 ± 1.71	77.30 ± 3.55	65.84 ± 2.86	86.61 ± 2.08	79.81 ± 2.17
w/o segmentation head	87.09 ± 0.88	86.10 ± 1.22	88.60 ± 1.81	/	/	/	/
w/o classification head	/	/	/	83.43 ± 0.98	74.20 ± 1.46	90.97 ± 0.59	85.18 ± 0.96

**Figure 5:**
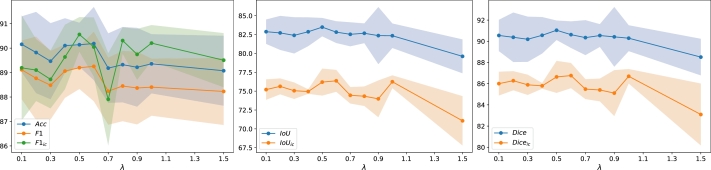
Performance metric variation across *λ*: (left) *Acc*, *F*1, *F*1_*ic*_; (center) *IoU*, *IoU*_*ic*_; (right) *Dice*, *Dice*_*ic*_.

Subsequently, we compared our proposed ResMTUNet with the head ablation variants, where the segmentation head and classification head were removed separately. Table 3 lists the performance metrics of ResMTUNet and two ablation variants on the segmentation and classification tasks in the test set. ResMTUNet consistently produces superior performance compared to the ablation variants across tasks. For the classification task, it outperforms the ablation variant with the segmentation head removed in terms of *F*1 and *Acc*; for the segmentation task, it achieves higher *IoU* and Dice than the ablation variant with the classification head removed. In addition, Fig. 5 illustrates the variation of various performance metrics of the proposed method with respect to the parameter *λ* on the BRACS dataset. From these curves, it is evident that the peaks of all performance metrics occur at *λ* values of 0.5 or 0.6, indicating the rationality of our hyperparameter selection.

**Figure 6:**
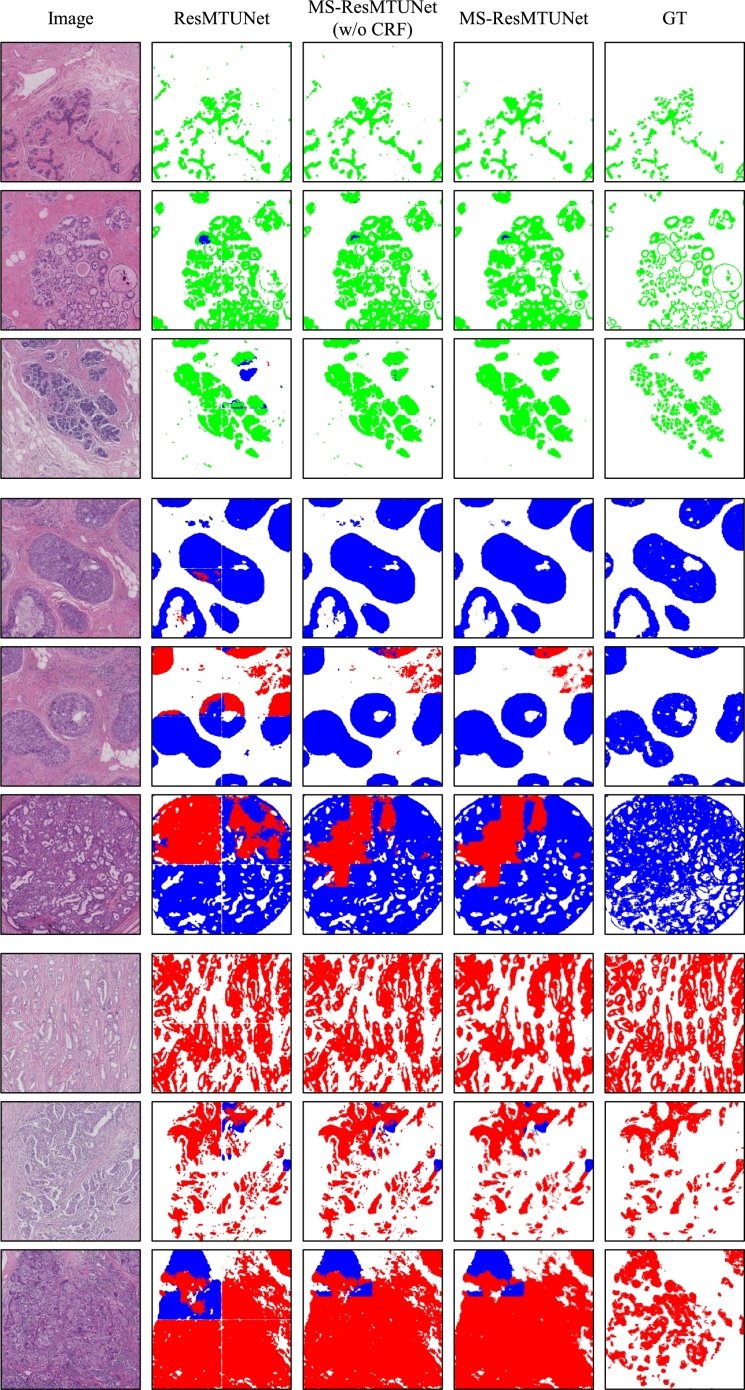
ROI-level predictions of different models. Row 1-3: Examples of ROI with IC. Row 4-6: Examples of ROI with DCIS. Row 7-9: Examples of ROI for normal breast tissue. Column 1: Original images. Column 2-4: Inference results by ResMTUNet, MS-ResMTUNet without CRF, and MS-ResMTUNet, respectively. Column 5: Ground truth.

To quantitatively assess the efficacy of our proposed method, we annotated and tested a set of 24 images from our internal dataset. Each image, sized at 3072×3072 pixels, represented instances of normal lobules, in-situ carcinoma, and invasive carcinoma (8 images per category). The evaluation metrics for the ResMTUNet and MS-ResMTUNet models are summarized in Table 4. Notably, the results reveal that MS-ResMTUNet consistently outperforms ResMTUNet across all metrics, highlighting the positive influence of multiscale information integration on overall segmentation performance. The subsequent introduction of CRF further refines the segmentation effectiveness, although its impact may vary for metrics. Fig. 6 visually demonstrates significant segmentation improvements achieved through the application of multiscale information and CRF. However, it's important to note that, in cases of substantial inference errors by ResMTUNet, the combined use of multiscale information and CRF may not entirely eliminate these errors.

**Table 4:** Evaluation Metrics for ResMTUNet and MS-ResMTUNet models.

	IoU${}_{normal}$	Dice${}_{normal}$	IoU${}_{dcis}$	Dice${}_{dcis}$	IoU${}_{ic}$	Dice${}_{ic}$	Avg. IoU	Avg. Dice
ResMTUNet	55.19	70.77	64.26	75.65	61.32	74.64	60.59	73.02
MS-ResMTUNet (w/o CRF)	57.74	72.92	73.76	83.92	**62.64**	**75.87**	64.38	77.57
MS-ResMTUNet	**58.77**	**73.71**	**74.62**	**84.58**	62.26	75.64	**65.22**	**77.64**

### 4.5 Inference on WSIs

**Figure 7:**
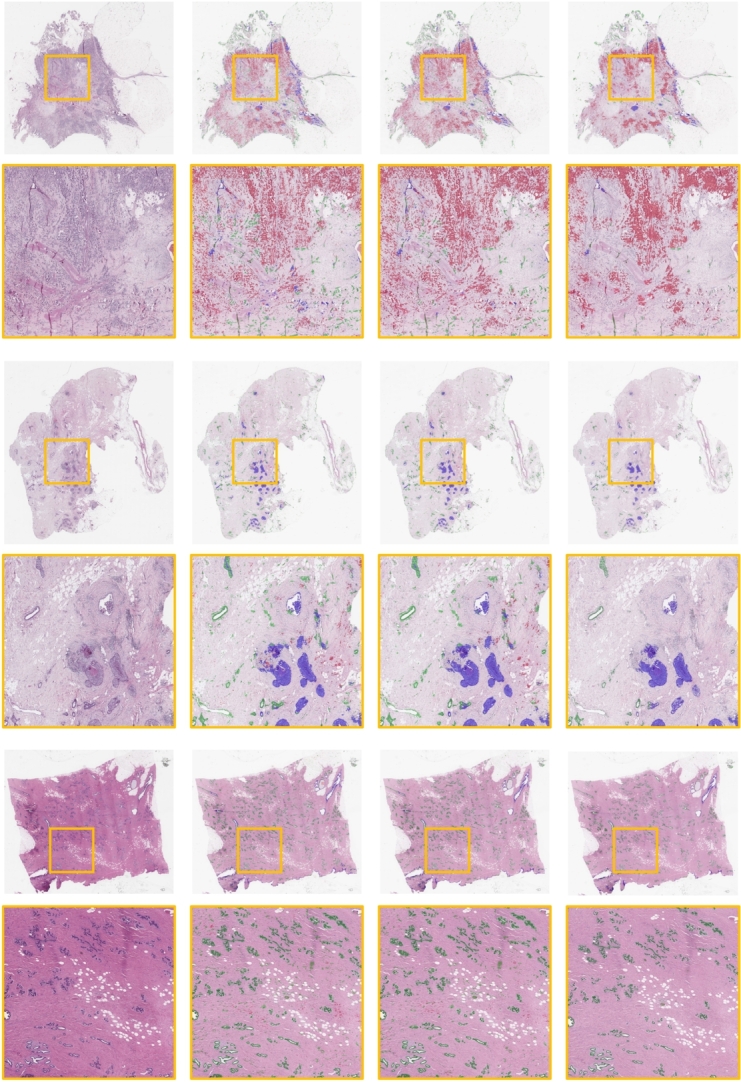
WSI-level predictions of different models. Row 1, 2: Examples of WSI with IC. Row 3, 4: Examples of WSI with DCIS. Row 5, 6: Examples of WSI for normal breast tissue. Column 1: Original images. Column 2-4: Inference results by ResMTUNet, MS-ResMTUNet without CRF, and MS-ResMTUNet, respectively.

To showcase the advantages of our proposed method for semantic segmentation in breast tissue WSIs, we compared it with original ResMTUNet model and multi-scaled ResMTUNet without post-processing via CRF. For qualitative results presentation, we selected three representative WSIs from the test set of BRACS. As shown in Fig. 7, the overall segmentation performance on the WSIs, as well as the local magnification of the segmentation results, demonstrates the potential of our proposed method in accurately segmenting invasive carcinoma regions. Our proposed method exhibits superior spatial consistency in semantic segmentation and avoids erroneously detecting invasive carcinoma areas in DCIS and normal samples.

### 4.6 Efficiency evaluation

To evaluate the efficiency of proposed model, we compared the performance of ResMTUNet with other segmentation models in terms of size and computational efficiency, as shown in Table 2. Specifically, ResMTUNet has a parameter count of 110.73M, indicating a higher complexity that allows it to capture more features and details. With a GFLOPs of 91.13, it requires relatively more computational power compared to other models during forward propagation.

In addition, we conducted an inference time comparison with two other variants: ResMTUNet and MS-ResMTUNet (without CRF). The results, presented in Table 5, show the average inference time in seconds for each model. ResMTUNet achieves the lowest inference time, averaging at 1.54±0.08 seconds. However, MS-ResMTUNet (without CRF) exhibits a slightly higher inference time of 2.43±0.10 seconds, which can be attributed to the utilization of the multi-scale feature fusion modules. On the other hand, MS-ResMTUNet demonstrates a significantly higher inference time of 17.23±0.39 seconds. This increase in computation time is primarily due to the reliance of the CRF layer on the CPU, which may limit the model's real-time performance. To overcome this limitation, we plan to explore the possibility of running the CRF on a GPU in the future. Overall, MS-ResMTUNet (without CRF) strikes a balance between accuracy and efficiency.

**Table 5:** Inference time comparison for ResMTUNet and MS-ResMTUNet models.

	Inference time (s)
ResMTUNet	1.54 ± 0.08
MS-ResMTUNet (w/o CRF)	2.43 ± 0.10
MS-ResMTUNet	17.23 ± 0.39

## 5 Discussion and conclusion

In this paper, we propose MS-ResMTUNet, a multi-scale input model based on a CNN-Transformer hybrid architecture for accurate invasive carcinoma recognition and segmentation on breast cancer WSIs. Experiments have demonstrated the superiority of our method over other competitive methods. In the future, transformer-based networks will play an important role in invasive carcinoma segmentation, laying a solid foundation for subsequent analysis of breast pathological slides.

Comparison of the proposed approach with existing approaches based on deep learning in Table 6 for qualitative assessment.

**Table 6:** Comparison with other existing approaches for breast tissue image segmentation.

Work	Dataset	Approach	Accuracy
DMMN [16]	Dataset-I: 32 WSIs; Dataset-II: 34 testing images	Architecture with multi-encoder, multi-decoder, and multi-concatenation	mIoU: 87% (dataset-I); 70.6% (dataset-II)
WSI-Net [17]	WSIs from Xiangya Hospital	A segmentation model (DeepLab) with a parallel classification branch	Cancer-mIoU: 61.36%; IDC-IoU: 68.59%
BM-Net [18]	BACH Dataset [61] (28 WSIs)	MobileNet-V3, Bilinear module	Average *Q*_*Score*_: 71%; 4 class accuracy (patch): 88%
IM-Net [62]	train/validation/testing images: 317/310/300	Semantic segmentation model	Dice: 83% (DCIS)
HookNet [63]	train/validation/testing WSIs: 50/18/18	Semantic segmentation model that combines context and details	Overall F1: 91%; IDC F1: 90%
**(MS-)ResMTUNet (proposed)**	BRACS (train/validation/testing images: 3657/312/570); SYSU test images: 24	Multitask learning, CNN-Transformer dual-branch, multi-scale, CRF	BRACS F1: 89.20% (overall), 90.56% (IC); BRACS IoU: 83.49% (overall), 76.20% (IC); BRACS Dice: 91.04% (overall), 86.64% (IC); SYSU IoU: 65.22% (overall), 62.26% (IC); SYSU Dice: 77.64% (overall), 75.64% (IC)

Ho et al. [16] presented a deep multi-magnification network that achieved an mIoU of 87% on dataset-I and 70.6% on dataset-II by incorporating multi-encoder, multi-decoder, and multi-concatenation techniques. Ni et al. [17] introduced WSI-Net, which combines a parallel classification branch with the DeepLab semantic segmentation model, achieving IoU scores of 61.36% in tumor regions and 68.59% in IDC regions. Huang et al. [18] developed a lightweight architecture that combines a bilinear structure and MobileNet-V3 network, demonstrating high performance with an accuracy of 88% and an average $Q_{score}$ of 71% on the BACH dataset [61]. Narayanan et al. [62] proposed IM-Net, which achieved a dice score of 83% for automated detection of individual DCIS ducts. Lastly, Rijthoven et al. [63] introduced HookNet, a semantic segmentation model that achieved an average F1 score of 91% overall and 90% for IDC on a 6-class segmentation task. These advancements contribute to the field of breast WSI analysis and hold promise for improved diagnosis and treatment.

In our approach, we achieved an overall F1 score of 89.20% and an F1 score of 90.56% on the IC, which is comparable to HookNet. For the BRACS dataset, we obtained an overall IoU of 83.49% and an IoU of 76.20% on the IC, surpassing the performance of most existing methods. While many other studies have also employed a multi-scale strategy, our work stands out by introducing the innovative Transformer-CNN dual-branch architecture, which demonstrates significant potential in breast tissue segmentation.

Although our research has made some progress, there are still areas that require further improvement. First, there is still room for improvement in the current model's performance. We aim to enhance our model's performance by exploring deep learning fusion strategies. One approach we will investigate is adaptively adjusting multi-scale weights to accommodate images with varying resolutions. Second, the current inference process of CRF running on CPU leads to slow processing time. To address this limitation, we plan to optimize the process for GPU acceleration in the future, significantly improving processing speed and efficiency. Furthermore, the proposed model has high computational requirements. We are considering knowledge distillation techniques to reduce computational requirements while maintaining performance, making our model more lightweight and accessible.

In the future, we will continue our research in two aspects. Firstly, using multi-center data can significantly enhance the model's performance. By integrating data from different medical centers, we can increase the diversity and coverage of our dataset, thereby boosting the model's generalization ability and accuracy. Such a multi-source dataset can more accurately reflect the diversity of the real world, making our model more reliable and practical. Secondly, integrating downstream tasks with invasive carcinoma segmentation models is crucial. Immunochemistry technology is an important component of modern pathology, providing pathologists with additional information about molecular expression in tumor tissues. Therefore, expanding our model to include immunohistochemistry data and integrating it with downstream tasks, such as Ki-67 index scoring, can further improve the model's application range and value.

## Ethics statement

Informed consent was not required for this study because there is no content that requires informed consent.

## Funding

The work was supported by 10.13039/501100001809National Natural Science Foundation of China (Grant Number: 61975089), 10.13039/501100010877Shenzhen Science and Technology Innovation Commission (Grant Number: KCXFZ20201221173207022), and Science and Technology Research Program of Shenzhen City (Grant Number: WDZC2020200821141349001, JCYJ20200109110606054).

## CRediT authorship contribution statement

**Yiqing Liu:** Writing – original draft, Visualization, Validation, Software, Investigation, Formal analysis, Data curation. **Huijuan Shi:** Writing – review & editing, Validation, Resources, Formal analysis. **Qiming He:** Writing – review & editing, Software. **Yuqiu Fu:** Visualization, Data curation. **Yizhi Wang:** Writing – review & editing. **Yonghong He:** Supervision, Conceptualization. **Anjia Han:** Supervision, Resources, Conceptualization. **Tian Guan:** Supervision, Project administration, Conceptualization.

## Declaration of Competing Interest

The authors declare that they have no known competing financial interests or personal relationships that could have appeared to influence the work reported in this paper.

## Data Availability

BRACS dataset can be downloaded from the following link: https://www.bracs.icar.cnr.it/. The in-house dataset will be made available on request.
